# Expanding the Versatility of Phage Display II: Improved Affinity Selection of Folded Domains on Protein VII and IX of the Filamentous Phage

**DOI:** 10.1371/journal.pone.0017433

**Published:** 2011-02-24

**Authors:** Geir Åge Løset, Norbert Roos, Bjarne Bogen, Inger Sandlie

**Affiliations:** 1 Centre for Immune Regulation, University of Oslo, Oslo, Norway; 2 Department of Molecular Biosciences, University of Oslo, Oslo, Norway; 3 Institute of Immunology, University of Oslo, Oslo, Norway; Science and Technology Facilities Council, United Kingdom

## Abstract

**Background:**

Phage display is a leading technology for selection of binders with affinity for specific target molecules. Polypeptides are normally displayed as fusions to the major coat protein VIII (pVIII) or the minor coat protein III (pIII). Whereas pVIII display suffers from drawbacks such as heterogeneity in display levels and polypeptide fusion size limitations, toxicity and infection interference effects have been described for pIII display. Thus, display on other coat proteins such as pVII or pIX might be more attractive. Neither pVII nor pIX display have gained widespread use or been characterized in detail like pIII and pVIII display.

**Methodology/Principal Findings:**

Here we present a side-by-side comparison of display on pIII with display on pVII and pIX. Polypeptides of interest (POIs) are fused to pVII or pIX. The *N*-terminal periplasmic signal sequence, which is required for phage integration of pIII and pVIII and that has been added to pVII and pIX in earlier studies, is omitted altogether. Although the POI display level on pIII is higher than on pVII and pIX, affinity selection with pVII and pIX display libraries is shown to be particularly efficient.

**Conclusions/Significance:**

Display through pVII and/or pIX represent platforms with characteristics that differ from those of the pIII platform. We have explored this to increase the performance and expand the use of phage display. In the paper, we describe effective affinity selection of folded domains displayed on pVII or pIX. This makes both platforms more attractive alternatives to conventional pIII and pVIII display than they were before.

## Introduction

Phage display is a leading technology for selection of binders with affinity for specific target molecules [1]. Libraries of polypeptides are created as fusions to phage coat proteins that are solvent exposed. The phage particle withstands physicochemical challenges that allow for highly diverse and versatile selection regimes [2], [3]. Thus, there is a clear-cut motivation for further increasing the performance and expanding the use of phage display.

**Figure 1:**
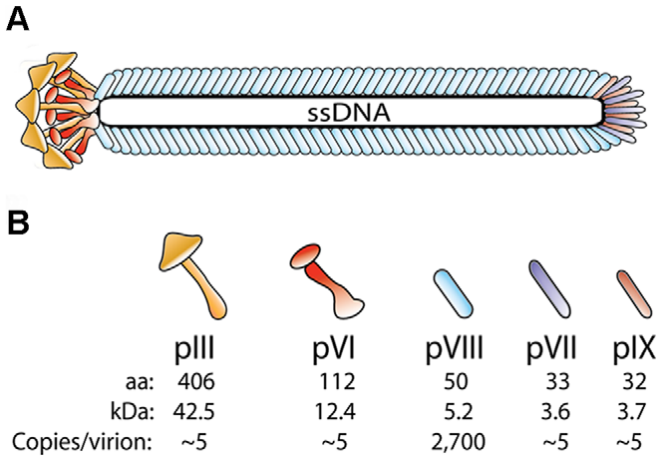
Schematic drawing of the filamentous phage structure. (**A**) The wt virion is made up of five structural proteins that coat a single stranded DNA genome of about 6.4 kb. (**B**) In the wt phage there is about 2,700 copies of pVIII and approximately 3–5 copies each of the four proteins pIII, pVI, pVII and pIX, which are found at each tip of the virion [4], [35]. The virion size depends on the genome size at approx. 2.3 nucleotides per pVIII, and hence the length of the particle changes as a function of genome length [36]. The theoretical MW of the mature capsid proteins were calculated from the sequence of VCSM13 (GenBank accession no.: AY598820).

The wt filamentous phage virions of M13, fd and f1 are composed of a small genome surrounded by a cylinder of coat proteins that measures about 1 µm in length and 8–10 nm in diameter, and has a total of about 2,700 copies of the major coat protein pVIII. In addition, the virion harbors approximately 3–5 copies of each of pIII, pVI, pVII and pIX; pIII and pVI on one virion tip and pVII and pIX on the other (Fig. 1) [4]. pIII is of particular importance since it is the critical component for the early events of *E. coli* host entry [5]. All are integral *E. coli* inner membrane proteins before virion assembly [6], but only pIII and pVIII are synthesized as precursors containing classical *N*-terminal signal sequences [4]. pVII and pIX appear to be synthesized without such signal peptides and consequently do not undergo post-translational processing [4].

Polypeptides have been displayed on all five structural proteins, but only pIII and pVIII display have gained widespread use. In both cases, the POI is normally placed *in-frame* between the *N*-terminal signal sequence and the mature form of the viral protein. POI-phage coat proteins may be encoded either in a phage genome or by a phagemid; in the latter case, complementation by a helper phage is needed to support virion production. Whenever a helper phage is utilized, it is an advantage during subsequent selection that the phagemid encoding the POI-fusion, and not the helper phage genome, is preferentially packaged in virions that display the POIs.

In pVIII display, there are polypeptide fusion size limitations that restrict its use primarily to small peptide fusions [7], [8]. The minor coat protein pIII tolerates larger fusions and pIII display performs better than pVIII display in high affinity selection [2], [9]. However, pIII is the critical viral protein for *E. coli* host infection, and POI fusion to pIII affects phage propagation, infectivity and causes repertoire bias [10], [11], [12]. Display on pVII or pIX might therefore be more attractive. In an early attempt to investigate pVII and pIX display, glutathione S-transferase was chosen as the POI and unsuccessful display was reported [6]. Later, successful display was shown to depend on a periplasmic signal sequence (pelB or ompA), being added to the *N*-terminus of the fusion protein [13]. Subsequent reports have therefore all utilized pelB-mediated periplasmic targeting of pVII and pIX fusions [14], [15], [16]. In an accompanying paper, we report that pVII displays a number of different peptide fusions without the need of an *N*-terminal signal sequence. The peptides (AviTag, FLAG and HIS_6_) were up to 17aa in length and have different charge and pI. This finding prompted us to compare display of large folded domains, such as single chain Fv (scFv) and single chain T cell receptor (scTCR), on pIII with display on pVII and pIX in the absence and presence of pelB. We demonstrate that the display level is higher on pIII than on pVII and pIX, and that the display level on pVII and pIX increases by including pelB. However, the presence of the signal sequence on pVII and pIX introduces a problem in that the helper phage genome, rather than the phagemid encoding the POI, is preferentially packaged in the virions after *E. coli* super-infection. In contrast, phagemid is preferentially packaged when it encodes the POIs fused to pVII and pIX without an *N*-terminal signal sequence. Importantly, both pVII and pIX display perform better than pIII display in affinity selection. This makes both an attractive alternative to conventional pIII and pVIII display for constructing libraries and subsequent selection of binders.

## Results

### Functional Display of Folded Domains on pVII and pIX

**Figure 2:**
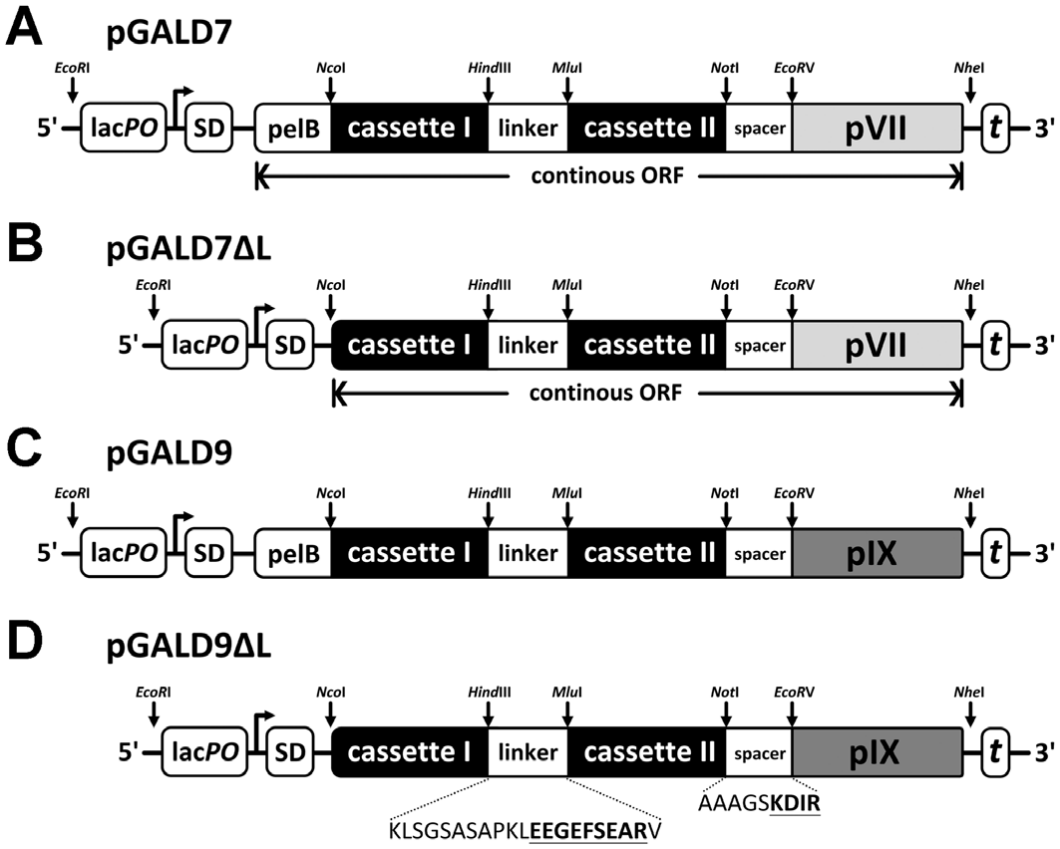
Schematic drawing of the pVII and pIX display phagemids. The full-length pIII of pSEX81 (GenBank accession no.: Y14584) was exchanged with either full-length pVII (*N*-MEQVADFDTIYQAMIQISVVLCFALGIIAGGQR-*C*) or pIX (*N*- MSVLVYSFASFVLGWCLRSGITYFTRLMETSS-*C*) retrieved from the M13K07 genome giving rise to pGALD7 (**A** and **B**) and pGALD9 (**C** and **D**), respectively. Moreover, both pGALD7 and pGALD9 were made such that the recombinant fusion was targeted either to the periplasm by a pelB signal sequence (**A** and **C**), or not (**B** and **D**). The phagemids can accommodate *in-frame* exogenous sequences (e.g. variable gene segments from Abs creating single chain Fv (scFv)) introduced through cassette exchange on *Nco*I/*Hind*III or *Mlu*I/*Not*I, respectively, thereby creating a continuous open reading frame (ORF). The two cassettes are connected by a 21aa synthetic linker containing the mAb Yol1/34 tubulin epitope (bold underlined) [18]. The heterologous polypeptide is fused to the capsid through a 9aa spacer containing a trypsin protease site (bold underlined). Unique restriction sites are indicated. Abbreviations: lacPO, lac promoter; SD, Shine-Dalgarno sequence; pelB, signal sequence of bacterial pectate lyase; t, T7 transcriptional terminator.

To study how pVII and pIX perform in phage display of folded domains, we tested display of three different folded domains and compared with conventional pIII display. Despite the fact that both pVII and pIX are expressed without *N*-terminal signal sequences and processing [4], they are inserted into and span the inner membrane of *E. coli* prior to virion incorporation. To study virion assembly, host cell viability as well as the ability of pVII and pIX to display folded domain fusions, a series of phagemid vectors were constructed with *N*-terminal POI fusions to pVII and pIX with or without the *N*-terminal signal sequence pelB (Fig. 2). The three POIs were two antibody scFvs (anti-phOx and anti-NIP) and a scTCR [17], which differ with regard to periplasmic expression efficiency in *E. coli,* ranging from very good (anti-phOx) to poor (scTCR) (Fig. S1).

**Figure 3:**
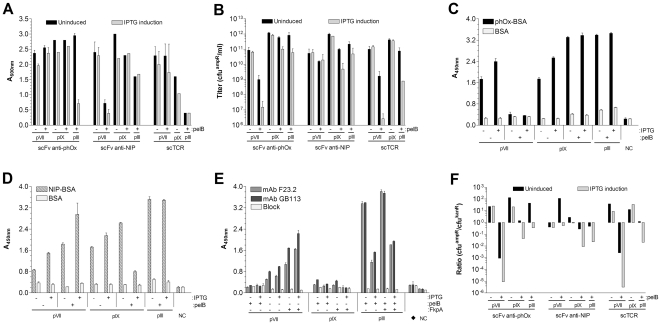
Evaluation of pIII, pVII and pIX mediated display of folded domains. (**A**) The cell density (A_600 nm_) of the individual phagemid packaging cultures was measured (as 1/4^th^ dilutions and the undiluted OD back-calculated) after ON growth at 30°C. (**B**) Phagemid titers in culture supernatants were determined by infectious titration. Transcription of the POI-capsid fusions, controlled by lacPO, was at basal level only after removal of repressor (glucose) from the growth medium (uninduced), or also induced by addition of 1 mM IPTG (IPTG induction). (**C** – **E**) Samples of cleared supernatant after phagemid rescue were used to assess antigen reactivity by phage capture ELISA. mAbs F23.2 and GB113 are conformation specific and clonotypic for the scTCR, respectively. NC is the anti-M13 detection Ab only and an irrelevant scTCR (7A10B2) was included as a negative control (♦). (**F**) The phagemid to helper phage ratio for the samples in **B** shown as cfu^ampR^/cfu^kanR^ based on infectious titration.

*E. coli* XL1-Blue was transformed with the various phagemids and host cell viability (measured as end culture cell density) and resulting phagemid titers were assessed following phagemid rescue. Transcription of the POI-capsid fusions was at *lac*PO basal level or after IPTG induction (Fig. 3A and B). In most cases, fusion to neither pVII nor pIX affected host cell viability, regardless of the presence of signal sequence. Whenever reduced host cell viability was observed, it did not translate into reduced production of virions. The virion yield was high throughout, except for in two cases: A marked reduction was observed upon pVII^pelB^ display of scFv anti-phOx and the scTCR.

The ability of the phage-borne POIs to bind their cognate ligands was investigated by semi quantitative phage capture ELISA as described in *Methods* (Fig. 3C–E). All POIs were found to bind specifically to antigen. Low phage yield reduced the signal. Thus, particularly low binding was observed for the scTCR, which is also poorly expressed as soluble protein (Fig. S1). We have previously demonstrated that over-expression of the periplasmic chaperone FkpA increases display of scTCRs when fused to pIII [17]. Here, we tested the effect of FkpA over-expression on pVII and pIX scTCR display (Fig. 3E), and demonstrate increased display on pVII, but not on pIX.

Virions produced after *E. coli* super-infection may have incorporated helper phage genome or phagemid encoding the POI fusion protein during assembly. The incorporation of phagemid is preferable since this increases the probability for successful affinity selection. We therefore investigated the genome content of the virions. The ratio of phagemid to helper phage containing particles was scored by measuring the titers of amp^R^ and kan^R^ colony forming units (cfu) in each case (Fig. 3F). A score value at or above 10^0^ indicates that at least 50% of the virion population contains the phagemid. For both pVII and pIX display this was always the case, also after IPTG induction. In the presence of signal sequence on pVII and pIX, on the other hand, it was not. Here, helper phage was preferentially packaged. For pIII, the majority of virions contained helper phage in one case (scFv anti-NIP) and phagemid in two cases (scFv-anti-phOx and scTCR). Whenever the helper phage was preferentially packaged, the effect was most dramatic after IPTG induction.

**Figure 4:**
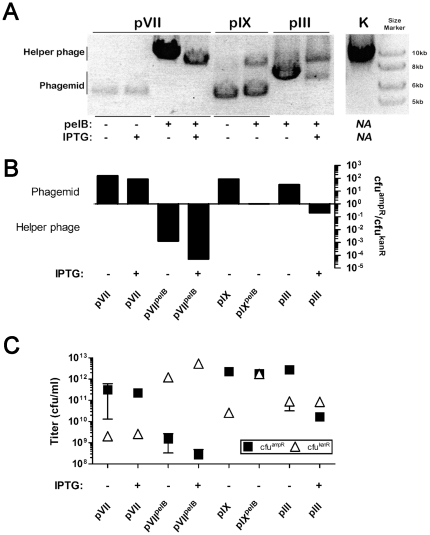
Agarose gel of virion ssDNA content and infectious titration analysis. (**A**) Equal volumes of PEG precipitated M13K07-rescued scFv anti-phOx display samples were separated on a 1% agarose gel, the virions denaturated and the ssDNA content visualized as described in *Methods*. The titer in-puts were not normalized; hence the band intensities directly reflect the ssDNA type and amount. The larger sized band is the helper phage genome, whereas the smaller sized bands are the phagemids. The M13K07 helper phage was included as a control (K). The actual phagemid/helper phage genome sizes are: pGALD7ΔL, 3679 bp; pGALD7, 3739 bp; pGALD9ΔL, 3679 bp; pGALD9, 3739 bp; pSEX81, 4882 bp and M13K07, 8669 bp. (**B**) The phagemid to helper phage ratio for the samples in **A** shown as cfu^ampR^/cfu^kanR^ based on infectious titration. (**C**) The primary infection titers in cfu/ml of the samples in **A**, which were used to determine the ratios depicted in **B**.

The large variation in phagemid to helper phage packaging ratios observed was surprising; hence to verify the result, rescued phagemid samples (scFv anti-phOx) were separated according to size on an agarose gel and the genomes visualized after phage particle denaturation in the gel (Fig. 4A). The approach was based on the fact that the virions accommodate to the size of the ssDNA they encapsulate. Virions that contain either phagemid encoding pVII or pIX fusions were 537±58 nm and 529±41 nm long, respectively. This is approximately 100 nm shorter than the length of the virions that contain the pIII fusion encoding phagemid (665±55 nm), and more than 700 nm shorter than the M13K07 helper phage genome containing virions (1256±138 nm) (Fig. S2). Distinct bands of ssDNA clearly demonstrated the presence of predominantly phagemid (pIII, pVII and pIX display), predominantly helper phage (pVII^pelB^ display) or a mixture of equal amounts of phagemid and helper phage (pIX^pelB^ display). Again, IPTG induction was shown to increase packaging of helper phage in pIII display. Subsequent infectious titration of the same samples almost perfectly mirrored the results from the ssDNA visualization (Fig. 4B and C). Furthermore, immunolabeling with anti-L_λ_ chain Ab and protein A gold in electron microscopy revealed that both short phagemid-containing and long helper phage-containing virions displayed fusion proteins (Fig. S3).

### Packaging Ratio May Influence Selection Efficiency

**Figure 5:**
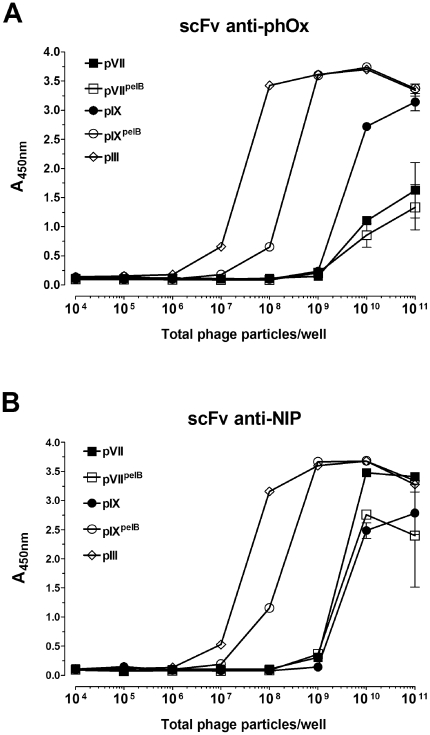
POI-capsid fusion display level determined as antigen specific binding. Serial dilutions of phagemid-rescued samples displaying either scFv anti-phOx (**A**), or scFv anti-NIP (**B**) were applied in a phage capture ELISA. Antigen-bound virions were detected by anti-M13-HRP and the data shown as a function of the number of virions applied per well. For all samples, the virion titer was determined by absorbance at A_268 nm_ and hence does not discriminate between phagemid or helper phage containing virions.

The actual display levels, defined as antigen specific binding in a phage capture ELISA with dilution series of virions, were then assessed for two specificities, scFv anti-phOx ((Fig. 5A) and scFv anti-NIP (Fig. 5B), and for each of the three coat proteins, pIII, pVII and pIX. Display of pVII and pIX fusion proteins were tested in the presence and absence of pelB mediated periplasmic targeting. For both specificities, the display on pVII and pIX were lower than on pIII. Periplasmic targeting improved pIX, but not pVII display.

We have previously shown that increased functional pIII display may translate into increased selection efficiency [18]. Here, we study the effect of the packaging ratio on selection using POI-display level-matched samples. Mixtures of virions that were helper phage-dominated or phagemid-dominated were subjected to a single round mock selection. Samples of scFv anti-phOx fused to pVII (dominated by phagemid virions) or pVII^pelB^ (dominated by helper phage virions) were used in antigen specific phage capture ELISA as above. The virion input was either high (10^10^ virions) or low (10^5^ virions). Following antigen binding and high stringency washing, captured virions were retrieved by *in-well* host cell infection, and the resulting cfu^ampR^ determined by selective propagation (Table 1). The results show that all samples were efficiently retrieved with high virion input, while only the pVII samples were retrieved with low virion input. The infectious (cfu^ampR^ and cfu^kanR^) virion input is given in Table S1. These data show that the lack of pVII^pelB^ retrieval at low virion input is likely due to the low number of phagemid containing virions in the input mixture (e.g. the actual phagemid pVII^pelB^ input in Experiment 2 was only 52 cfu^ampR^).

**Table 1:** Infectious output (cfu^ampR^) after monoclonal mock selection.

Experiment no.*	Display type	Input: 10^10^ virions**	Input: 10^5^ virions**
**1**	pVII	∞	6
**1**	pVII^pelB^	∞	0
**2**	pVII	∞	9
**2**	pVII^pelB^	1728	0

*Two independent experiments were performed, both using freshly prepared samples.

**Determined by A_268 nm._

### Improved Affinity Selection of Binders Displayed on pVII and pIX

**Figure 6:**
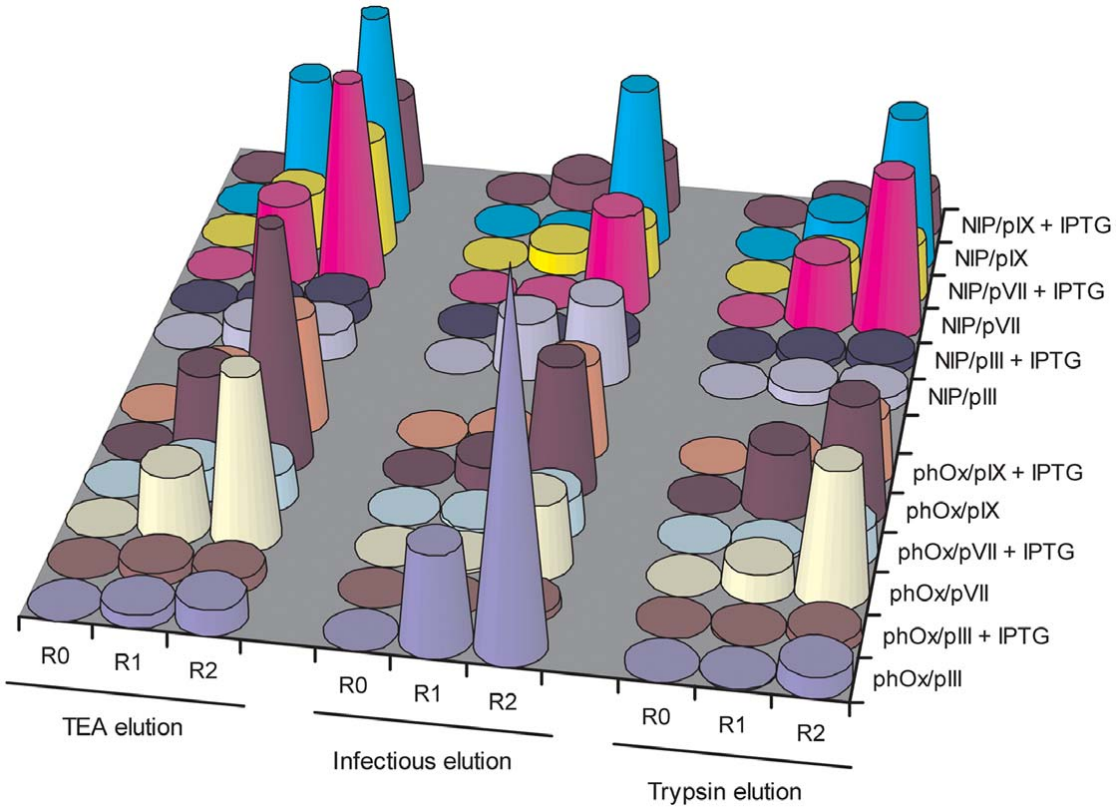
Antigen-specific enrichment in affinity selection depends on capsid display scaffold. Two rounds of affinity selection were performed using two different scFv specificities displayed on either of pIII, pVII or pIX. In each library, a total of 12, the specific scFv was spiked into a large background of virions (1∶10^7^) as described in *Methods*. All selections were done in parallel on both antigens (phOx-BSA and NIP-BSA) employing three different virion elution conditions following antigen binding; high pH (TEA), direct *in-well* infection, or proteolytic antigen-virion disruption (trypsin). Following amplification, equal volumes of virion-containing culture supernatants from selection round 1 and 2 were assessed for antigen reactivity by phage capture ELISA. Round 0 corresponds to the spiked input of 1×10^10^ cfu^ampR^. To estimate maximum possible response (100% enrichment of the specific scFv), culture supernatants from the pure specific virions that were spiked into the libraries were used as reference for the corresponding selection. The results are given as fraction of the maximum, indicated by cone shape. Importantly, the virion titers of all supernatants were roughly equal (*data not shown*).

To elucidate how pVII and pIX display perform in affinity selection, we again compared them with conventional pIII display. Virions were produced in the presence or absence of IPTG induction, the POIs being either scFv anti-phOx or anti-NIP, respectively. Thus, a total of 6 phage populations were evaluated for the two antigens phOx- and NIP-BSA. In each case, the antigen specific virions were mixed with the specificity irrelevant virion at a ratio of 1∶10^7^. The two scFvs do not cross-react (*data not shown*). Two rounds of affinity selection were then carried out. We used three different elution strategies; high pH, proteolysis with trypsin or direct infection. Enrichment was analysed by comparing the signals from the unselected mock library (R0) with the outputs of the first (R1) and second (R2) round of selection using an antigen specific phage capture ELISA (Fig. 6). The results showed that pVII and pIX perform better than pIII in affinity selection in all but one case, namely phOx selection using elution by direct infection. The standard pIII display route employing high pH (TEA) or proteolytic (trypsin) elution exhibited poor enrichment compared to pVII and pIX. Selection was more efficient without than with IPTG induction, independently of display route and elution conditions, and the negative effect of IPTG induction was most severe for pIII display route.

## Discussion

Phage display on pIII and pVIII is based on signal sequence dependent translocation of the POI-capsid fusion from the cytosol to the periplasm of the *E. coli* host. We show here display and efficient affinity selection using two alternative capsid proteins, pVII and pIX. In contrast to earlier reports, this was achieved without the aid of a conventional post-translationally cleaved *N*-terminal signal sequence. Both pVII and pIX lack known signal sequences and are yet inserted into and span the inner *E. coli* plasma membrane prior to virion incorporation without post-translational processing [4], [6]. The discrepancy between the current study and an earlier report [6] regarding the feasibility of genomic pVII fusion protein display may at least partly be due to the nature of the fusion protein used previously, namely glutathione-S-transferase, which is a globular protein that readily folds and dimerizes in the cytosol [19]. This may have hampered its periplasmic targeting.

Two different scFvs and one scTCR were displayed on pIII as well as on pVII and pIX in the absence or presence of a periplasmic signal sequence. All were expressed from phagemids. A recent report of signal sequence mediated genomic scFv-pVII display described reduced virion titers [20]. Here we extend this observation, as viral titers were reduced when two of three POIs (scFv anti-phOx and scTCR) were displayed on pVII in the presence, but not in the absence, of signal sequence.

We found the display level to vary in virion populations with the POIs displayed on pIII, pVII or pIX. In all cases, it was higher on pIII than on pVII and pIX. The addition of an *N*-terminal signal sequence increased display on pIX, but not on pVII. However, helper phages were preferentially packaged after super-infection. Display may well differ between the two populations of phages (either long, containing helper phages, or short, containing phagemid), and in a first step, we were able to confirm by electron microscopy that both long and short particles were able to display POI-fusions. The relative display levels on the two populations are not known. However, specific antigen binding for pIX^pelB^ and VII^pelB^ was seen despite the fact that both had highly unfavorable phagemid to helper phage ratios.

In a selection regime “the retrievable virions”, those that contain phagemid in the input population, coexist with the virions that contain helper phage genomes in a single mixed population. When specific binders are present in low numbers, or are expressed at low levels, as part of a library with large diversity, consistently high yields of phagemid titers are crucial for successful retrieval. Given the solubility threshold before spontaneous virion precipitation at about 10^13^ virions/ml, the actual phagemid titer of samples with unfavorable phagemid to helper phage ratios may fall below a threshold that ensures their representation. Thus, antigen-specific library members exhibiting preferentially helper phage packaging may be lost during selection both through unsuccessful competition for available antigen, the lack of a POI encoding genome, or a combination of both. This is illustrated by the results from a single round mock selection procedure, designed to mimic varying library complexity. The virion input was constant, but the number of phagemid containing particles within the samples differed, depending on the packaging preferences (phagemid or helper phage) in each case. Notably, all samples with low phagemid titer consistently exhibited high helper phage titers, which in effect yielded high total virion content (determined by absorbance at A_268 nm_). Samples of pVII and pVII^pelB^ virions at high and low titers were investigated for antigen specific retrieval. While one sample was helper phage dominated (pVII^pelB^), the other was phagemid dominated (pVII). Indeed, the helper phage dominated pVII^pelB^ sample was lost, while virions from the pVII sample were retrieved, after selection carried out with low virion input.

The scTCR chosen as one of three POIs studied in the present work is displayed poorly as fusion to all viral proteins investigated [17]. In general, display constraints may be alleviated by manipulating the periplasmic chaperone environment [21], altering the periplasmic targeting propensity [22], or route [23]. Here we show that over-expression of the periplasmic protein FkpA increased display on pVII, as well as on pIII as demonstrated previously [17]. Surprisingly, pIX display was not improved. The reason for this is not known. Nonetheless, the data show that both pVII and pIX functionally display all three POIs despite their varying soluble expression characteristics, and this is important when considering future library design [24].

Affinity selection of specific binders was highly efficient using both pVII and pIX fusions without an *N*-terminal signal sequence. In five out of six cases, it was more efficient than pIII mediated affinity selection. Thus, the low display levels observed for pVII and pIX did not translate into poor selection. Small differences in display level observed between pVII and pIX did not translate into differences in selection efficiency. Rather, the preferential phagemid packaging contributed to efficient retrieval.

The lower display levels on pVII and pIX are not necessarily a disadvantage. On the contrary, low level display may be desirable during selection of strong binders from complex libraries due to true monovalent display [10], [25]. Furthermore, by leaving pIII unaltered and fully solvent exposed, virion rescue following selection may be performed effectively without breaking the virion-antigen bond, making the elution step redundant and speeding up high through put protocols. The latter may also facilitate the isolation of high affinity binders, the elution of which may be resistant to a variety of strategies [26]. Combined with complete absence of pIII-mediated immunity effects [12] and heterogeneous signal sequence cleavage [7], [27], [28] that cause random library repertoire bias, the pVII and pIX display platforms may outperform pIII during affinity selection. Thus, pVII and pIX display extends the utility of phage display.

## Materials and Methods

### Plasmids, bacterial strains, phage and materials

The phOx-BSA and pSEX81 phagemid vector (GenBank accession no.: Y14584) harboring a phOx-BSA specific affinity matured human scFv was kindly provided by Affitech Research AS (Oslo, Norway). The pSEX and pFKPDN phagemids harboring either the scTCR Vαβ4B2A1 or 7A10B2 have been described previously [17]. The pHOG21 derivative pSG1 harboring a NIP-BSA specific murine scFv were constructed in-house (*unpublished*). The *E. coli* strain XL1-Blue (*recA1 endA1 gyrA96 thi-1 hsdR17 supE44 relA1 lac* [F' *proAB lacI* ^q^ *Z*Δ*M15* Tn*10* (Tet^r^)] and the M13K07 helper phage were purchased from Stratagene (LaJolla, CA, USA) and GE Healthcare (Uppsala, Sweden), respectively. All restriction enzymes were purchased from New England Biolabs (Ipswich, MA, USA). DNA oligos were purchased from MWG Biotech AG (Ebersberg, Germany). Pfu Ultra and Phusion DNA polymerases were purchased from Stratagene (LaJolla, CA, USA) and Sigma-Aldrich (Oslo, Norway), respectively. The NIP-BSA conjugate was prepared as described [29]. The anti-M13-HRP Ab was purchased from GE Healtcare (Uppsala, Sweden). The F23.2 mAb was a kind gift from Dr. Uwe D. Staerz (Department of Medicine, National Jewish Medical and Research Center, Denver, USA) and the GB113 mAb [30] was purified from cell supernatant on protein G-sepharose (GE Healtcare, Uppsala, Sweden). Rabbit anti-human L_λ_ chain Ab was from DakoCytomation (Glostrup, Denmark) and fish skin gelatine, protein A gold (φ10 nm) solution, as well as Triethylamine (TEA) and bovine serum albumin (BSA), were from Sigma-Aldrich (Oslo, Norway). Trypsin/EDTA was purchased from BioWhittaker (Lonza Group Ltd., Visp, Switzerland). All media and buffers were prepared essentially as described [31].

### Design and construction of pVII and pIX display phagemids

The pelB signal sequence (*N*-MKYLLPTAAAGLLLLAAQPAMA-*C*) encoding stretch of the pSEX81 (GenBank accession no.: Y14584) scFv anti-phOx phagemid vector was removed by QuikChange™ *in vitro* mutagenesis (Stratagene (LaJolla, CA, USA)). The mutated region was PCR amplified and re-cloned into corresponding region in the mother plasmid using standard techniques and confirmed by DNA sequencing. This step completely removed the pelB signal sequence encoding portion, but preserved the start codon and its relative position towards the lac*PO* and Shine-Dalgarno sequence important for normal transcription and translation, as well as adding only one Ala residue before the exogenous sequence defined by the *Nco*I/*Not*I sites found in the original pSEX81. The new construct was denoted pSEX81ΔL. Secondly, the pVII and pXI encoding sequences were PCR amplified from M13K07 and moved into both the pSEX81, and pSEX81ΔL phagemids on the compatible *EcoR*V/*Nhe*I sites, thereby exchanging the pIII encoding region in both and resulting in a *N*-terminal *in-frame* fusion to either pVII (*N*-MEQVADFDTIYQAMIQISVVLCFALGIIAGGQR-*C*) or pIX (*N*- MSVLVYSFASFVLGWCLRSGITYFTRLMETSS-*C*) of the upstream *Nco*I/*Not*I-defined cassette. The new constructs were confirmed by DNA sequencing and denoted pGALD7, pGALD7ΔL, pGALD9 and pGALD9ΔL, respectively (Fig. 2). To switch the scFv anti-phOx unit in the various phagemids described above, with the scFv anti-NIP unit from pSG1, or the scTCR from pFKPDN, this was done as *Nco*I/*Not*I defined cassette exchange using standard techniques. All phagemids described herein were introduced into *E. coli* XL1-Blue by electroporation using standard techniques. Primer sequences and GenBank accession numbers for the constructs are listed in Tables S2 and S3, respectively.

### Virion production

Phagemid rescue from *E. coli* XL1-Blue was done essentially as described [32], except for the addition of 1 mM (final concentration) isopropyl-β-D-thiogalactoside (IPTG) where indicated. Virion assembly was monitored either by infectious spot titration as described [11], or as total virion titer by optical density at A_268 nm_ [2]. Where applicable, the virions were purified and concentrated by PEG/NaCl precipitation as described [33], and resuspended in PBS, pH 7.4.

### Agarose gel electrophoresis of intact virions

Volumes of 25 µl PEG precipitated phage samples were separated for 3 h at 60 V at RT on a 1% agarose gel in Tris-Acetate-EDTA (TAE) buffer. Notably, the samples were not titer normalize. The gel was then incubated with 0.2 M NaOH for 1 h, rinsed with dH_2_O water and neutralized with 1 M Tris–HCl, pH 7.0 for 15 min. The ssDNA bands from denatured phage and phagemid virions were visualized by 30 min incubation with SYBR Safe DNA gel stain (Invitrogen, Carlsbad, CA, USA) solution (1∶10,000 v/v in dH_2_O) and imaged on a Bio-Rad Gel Doc 2000 work station (Bio-Rad, Hercules, CA, USA).

### Phage capture ELISA

In ELISA, the various antigens (Abs, NIP-BSA and phOx-BSA) were absorbed to MaxiSorp™ microtiter plate wells (Nunc, Roskilde, Denmark) in concentrations from 2.5 to 5 µg/ml in PBS, pH 7.4 overnight at 4°C. The wells were blocked with PBSTM (PBS supplemented with 0.05% v/v Tween 20 and 4% w/v skim milk) for 1 h at room temperature (RT), virion preparations where then added and allowed to react for 1 to 2 h at RT before captured virions were detected with anti-M13-HRP (1∶5,000) for 1 h at RT. A 3× washing step with PBST (PBS supplemented with 0.05% v/v Tween 20) was applied between each incubation step. The wells were developed with ABTS substrate and the absorbance read at A_405 nm_. Alternatively, the wells were developed with TMB soluble (Merck KGaA, Darmstadt, Germany), stopped with 1 M HCl, equilibrated and the absorbance read at A_450 nm_.

Phage capture ELISA was followed by *E.coli* infection and the cfu^ampR^ estimated as follows: phOx-BSA or BSA (5 µg/ml) were used as coat. Blocking was as described above. Samples of 10^10^ or 10^5^ virions (determined by A_268 nm_) were added per well and allowed to react for 2 h at RT before the wells were washed ×9 in PBST followed by ×5 in dH_2_O. Volumes of 200 µl log-phase (A_600 nm_ ∼0.5) *E. coli* XL1-Blue (≥5×10^7^ cells) were added to each well and incubated for 35 min at 37°C before plating on LB-amp. Resulting cfu^ampR^ were counted after overnight incubation at 37°C.

### Electron microscopy and POI fusion immunolabeling

Samples normalized to 1×10^10^/ml were agitated for 10 sec on a vortex shaker. 5 µl drops of each sample were applied to a clean surface and 100 mesh copper grids placed on top of the drops in order to absorb the particles. The grids were then washed for 10 min on a total of 3 drops of PBS pH 7.4 followed by a 10 min wash on 6 drops of triple distilled water. The samples were subsequently stained with a 2% aqueous uranyl-acetate solution for 3 min and air-dried before observation in a Philips CM100 transmission electron microscope at 80 kV. Images were recorded at a magnification of 25, 000× and a measuring grid was overlayed. Intersections with the grid were counted and the length of each particle was calculated using π/4 * I * d (I being the number of intersections and d being the distance between the grid lines) [34].

For immunolabeling of POI-capsid fusions, samples normalized to 1×10^10^/ml were agitated for 10 sec on a vortex shaker. 5 µl drops of each sample were then applied onto parafilm slits and 100 mesh copper grids placed on top of the drops in order to absorb the particles. They were then washed for 5 min on a total of 4 drops of PBS pH 7.4. The grids were subsequently placed on drops of rabbit anti-human L_λ_ chain Ab diluted 1∶25 in 1% fish skin gelatine w/v PBS for 30 min and washed in 5 drops of PBS pH 7.4 for a total of 10 min. After the washing step the grids were exposed to a protein A-gold (φ10 nm) solution for 20 min, washed on 3 subsequent drops of PBS (for a total of 5 min) followed by 5 drops of dH_2_O (for a total of 10 min). Staining and microscopy was performed as described above.

### Spiked phOx-/NIP-BSA selection

Fresh virion samples were prepared, either with or without 1 mM IPTG induction, PEG precipitated and titrated as described. The antigen specific entity was then spiked into an irrelevant background at a 1∶10^7^ level giving a known diversity of 10^7^, corresponding to a medium sized combinatorial library. For NIP-BSA selection, the scFv anti-NIP was spiked into the scFv anti-phOx counterpart and vice versa. The initial input was 1×10^10^ cfu^ampR^ resulting in a complexity level of 10^3^ in panning round 1 for all the 12 spiked libraries. Briefly, antigen was immobilized on MaxiSorp™ microtiter plate wells (Nunc, Roskilde, Denmark) in triplicates on the same plate using 100 µl volumes of 1 µg/ml and 0.1 µg/ml for panning round 1 and 2, respectively. Prior to panning, the wells were blocked with PBSTM for 1–2 h at RT, before 100 µl of the respective pre-blocked (in PBSTM) virion preparations where added and allowed to react for 1.5 h at RT with agitation. The wells were washed 9× in PBST followed by 5× in dH_2_O using a microtiter washer before antigen-bound virions (in triplicate wells) were eluted by either **1**) adding 100 µl/well of 100 mM TEA (pH 12) for 5 min at RT followed by neutralization by transfer to fresh well containing 100 µl/well Tris-HCl, pH 6.8; **2**) adding 100 µl/well Tryspin/EDTA for 10 min/RT followed by transfer to fresh wells; **3**) adding 200 µl/well log-phase (A_600 nm_ ∼0.5, corresponds to ≥5×10^7^ cells) *E. coli* XL1-Blue for 30 min at 37°C with agitation, followed by transfer to 10 ml pre-warmed YT-TAG (2× YT containing 30 µg/ml tetracycline, 100 µg/ml ampicillin and 0.1 M glucose) supplemented with AviTag-pVII modified M13K07 helper phage at MOI10. The incubation was continued for 15 min at 37°C with low agitation followed by 30 min at 37°C with high agitation. In parallel, the TEA and Trypsin eluted samples were used to infect log-phase *E. coli* XL1-Blue cultures in 9 ml YT-TAG, incubated with low agitation for 15 min at 37°C, before 1 ml YT-TAG supplemented with AviTag-pVII modified M13K07 helper phage at MOI10 was added. The incubation was continued for 15 min at 37°C with low agitation followed by 30 min at 37°C with rigorous agitation. All samples were then were centrifuged 3000-*g*/10 min/RT, the supernatants discarded and the pellets gently resuspended in 10 ml pre-warmed 2× YT containing 100 µg/ml ampicillin and 50 µg/ml kanamycin. The appropriate samples were supplemented with 1 mM IPTG (final concentration) and all samples incubated ON at 30°C with rigorous agitation. The day after, the cultures were centrifuged 4000-*g*/10 min/RT and the supernatant sterile filtered into fresh 15-ml tubes trough 0.2 µl filters. These supernatants where then channeled into next round of panning as described, using 50 µl volumes/sample corresponding to an input of at least 10^9^ cfu^ampR^/sample. Following the 2^nd^ round of selection the virion containing supernatants were channeled into an antigen-specific ELISA as described above.

## Supporting Information

Figure S1 Soluble scFv and scTCR expression profiles. *E. coli* XL1-Blue cells harboring pHOG21 constructs encoding the three single chain versions were grown and samples prepared essentially as described [37]. Briefly, 5 ml expression end cultures were normalized by A_600nm_ to represent the same number of cells and separated into the medium (M), periplasmic (P) and cytosolic (C) fractions. Equal volumes of each fraction were separated by 12% SDS-PAGE, blotted onto PVDF membranes and recombinant protein detected with an antibody specific for the *C*-terminal myc-tag. Expression was done for 6h (**A**), or ON (**B**), before sub-cellular fractionation. The phage display selected human scFv anti-phOx exhibits a highly favorable expression pattern, and can be found both in the periplasm and medium. The murine hybridoma-derived scFv anti-NIP can be obtained from the periplasm, but exhibits low to no secretion to the medium. The murine scTCR 4B2A1 is found exclusively as aggregated material in the cytosol, but exhibits only modest toxicity effects on the host cells. Notably, the variable gene segments in the three constructs (which also apply to the phage displayed versions) are connected by different synthetic linkers as follows: scFv anti-phOx (*N*-SGSASAPKLEEGEFSEARV-*C*), scFv anti-NIP (*N*-GGGGSGGGGSGGGGS-*C*) and scTCR (*N*-KLSGSASAPKLEEGEFSEARV-*C*).
(TIF)

Figure S2 The virion length is proportional to the size of the genome it encapsulates. (**A**) Transmission electron micrographs showing negative stained virions of long, intermediate and short morphology at a magnification of 25, 000x. (**B**) Blind selections of each virion type were measured by transmission electron microscopy and depicted as the mean (n = 30 - 50) ± SD of the population. The theoretical virion length was estimated based on genome sizes using the formula [(1.435 Å × bp) + 175 Å] [38]. Genome size to virion length correlation was determined using linear regression and shown to be virtually linear. Thus, the phagemids encoding pVII and pIX fusion proteins are encapsulated in very small virions (pGALD7ΔL and pGALD9ΔL: 537±58 and 529±41 nm, respectively), which is about 100 nm shorter than for a pIII phagemid (pSEX81: 665 ± 55 nm), almost 800 nm shorter than for a helper phage, M13K07, and 900 nm shorter than for a genomic pIII display system (fUSE5: 1413±138 nm).
(TIF)

Figure S3 Immunolabeling of scFv displayed on pVII and pIX. Transmission electron micrographs showing negatively stained phagemid-derived samples displaying the scFv anti-phOx on either pVII or pIX. The scFv fusion was immunolabeled with φ10nm gold-particles against the human L_λ_-chain region as described in *Methods.* ***Upper left***, signal sequence-dependent pVII display with the pGALD7-rescued phagemid (phagemid:helper phage, 1∶10^7^). ***Lower left***, signal sequence-dependent pIX display with the pGALD9-rescued phagemid (phagemid:helper phage, 1∶1). ***Upper right***, signal sequence-independent pVII display with the pGALD7ΔL-rescued phagemid (phagemid:helper phage, 20∶1). ***Lower right***, signal sequence-independent pIX display with the pGALD9ΔL-rescued phagemid (phagemid:helper phage, 261∶1). As depicted in Fig. S2, the phagemid and helper phage virions can easily be distinguished based on either short or long morphology, respectively. Yet only the phagemid encodes the scFv, both short and long virions display the fusion protein. The samples exhibit strong variation in phagemid to helper phage rations (determined by infectious titration) depending on capsid and presence or absence of the signal sequence on the fusion protein. Scale bar: 200nm.
(TIF)

Table S1 A_268nm_ vs. infection titer input in the “monoclonal” mock selection.
(DOC)

Table S2 Oligonucleotide sequences.
(DOC)

Table S3 GenBank accession numbers.
(DOC)
