# Supplementary figures and images for: Expanding the Versatility of Phage Display II: Improved Affinity Selection of Folded Domains on Protein VII and IX of the Filamentous Phage
Source: PLoS One. 2011 Feb 24;6(2):e17433. doi: 10.1371/journal.pone.0017433 (PMC3044770; doi:10.1371/journal.pone.0017433)

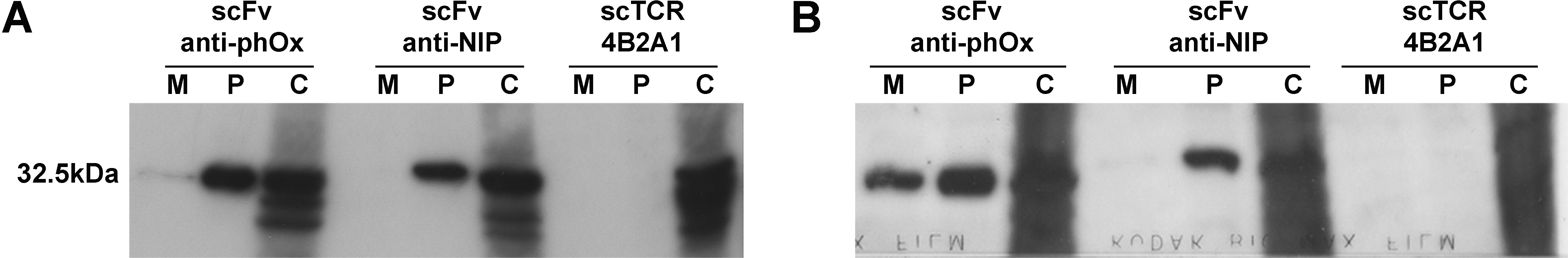

Supplement: Figure S1 — Soluble scFv and scTCR expression profiles. E. coli XL1-Blue cells harboring pHOG21 constructs encoding the three single chain versions were grown and samples prepared essentially as described [37]. Briefly, 5 ml expression end cultures were normalized by A600nm to represent the same number of cells and separated into the medium (M), periplasmic (P) and cytosolic (C) fractions. Equal volumes of each fraction were separated by 12% SDS-PAGE, blotted onto PVDF membranes and recombinant protein detected with an antibody specific for the C-terminal myc-tag. Expression was done for 6h (A), or ON (B), before sub-cellular fractionation. The phage display selected human scFv anti-phOx exhibits a highly favorable expression pattern, and can be found both in the periplasm and medium. The murine hybridoma-derived scFv anti-NIP can be obtained from the periplasm, but exhibits low to no secretion to the medium. The murine scTCR 4B2A1 is found exclusively as aggregated material in the cytosol, but exhibits only modest toxicity effects on the host cells. Notably, the variable gene segments in the three constructs (which also apply to the phage displayed versions) are connected by different synthetic linkers as follows: scFv anti-phOx (N-SGSASAPKLEEGEFSEARV-C), scFv anti-NIP (N-GGGGSGGGGSGGGGS-C) and scTCR (N-KLSGSASAPKLEEGEFSEARV-C). (TIF) [file pone.0017433.s001.tif]

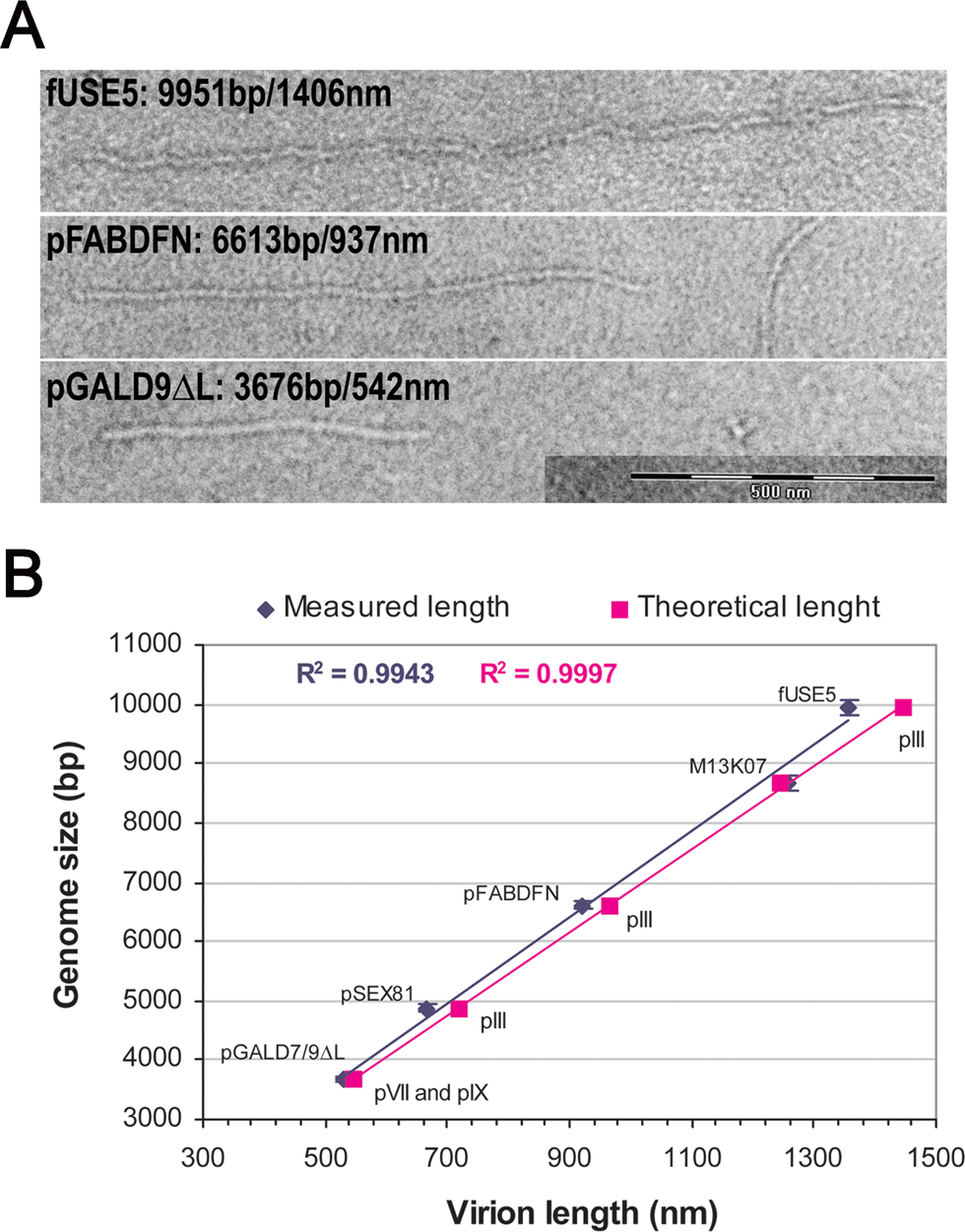

Supplement: Figure S2 — The virion length is proportional to the size of the genome it encapsulates. (A) Transmission electron micrographs showing negative stained virions of long, intermediate and short morphology at a magnification of 25, 000x. (B) Blind selections of each virion type were measured by transmission electron microscopy and depicted as the mean (n = 30 - 50) ± SD of the population. The theoretical virion length was estimated based on genome sizes using the formula [(1.435 Å × bp) + 175 Å] [38]. Genome size to virion length correlation was determined using linear regression and shown to be virtually linear. Thus, the phagemids encoding pVII and pIX fusion proteins are encapsulated in very small virions (pGALD7ΔL and pGALD9ΔL: 537±58 and 529±41 nm, respectively), which is about 100 nm shorter than for a pIII phagemid (pSEX81: 665 ± 55 nm), almost 800 nm shorter than for a helper phage, M13K07, and 900 nm shorter than for a genomic pIII display system (fUSE5: 1413±138 nm). (TIF) [file pone.0017433.s002.tif]

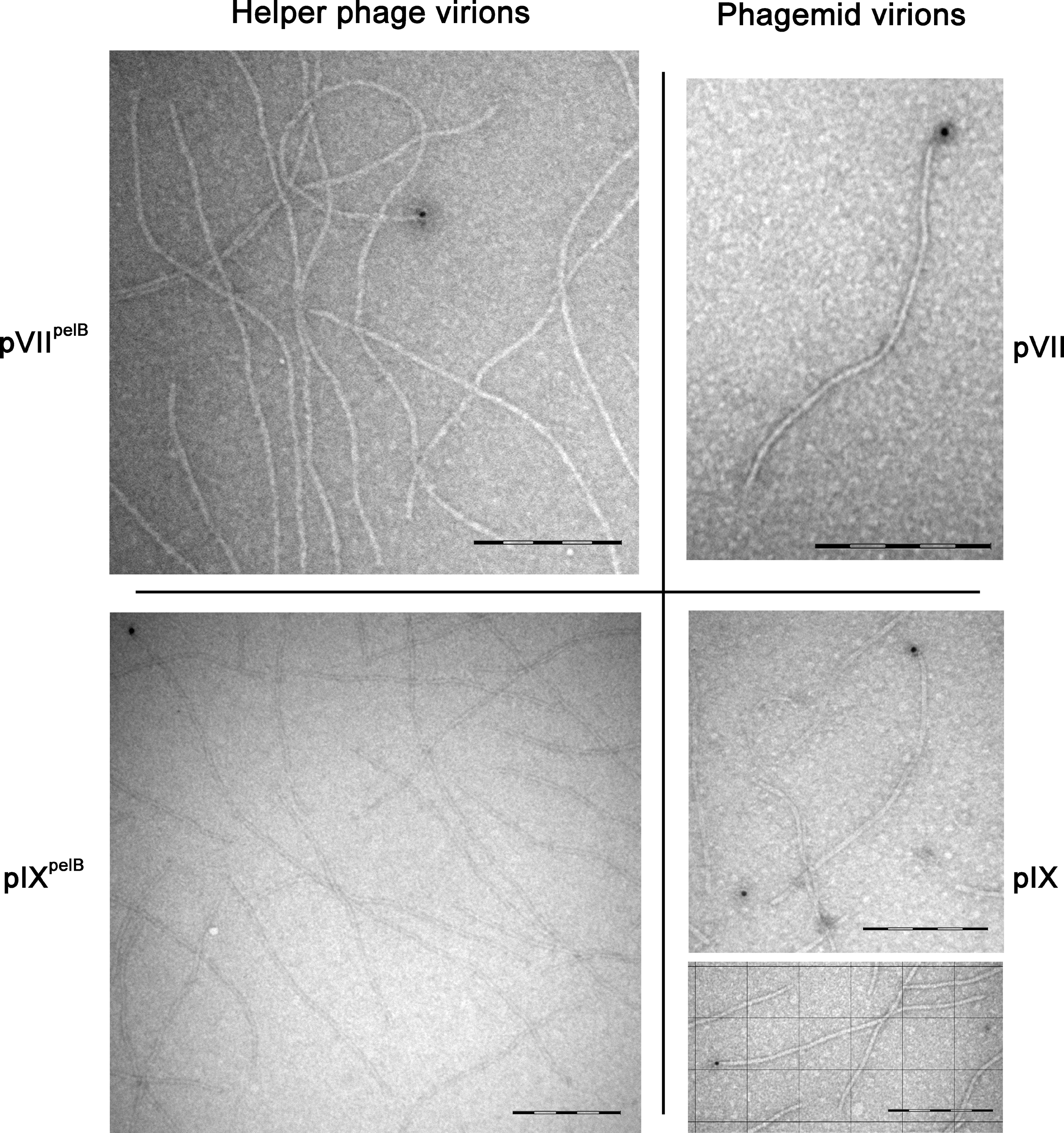

Supplement: Figure S3 — Immunolabeling of scFv displayed on pVII and pIX. Transmission electron micrographs showing negatively stained phagemid-derived samples displaying the scFv anti-phOx on either pVII or pIX. The scFv fusion was immunolabeled with φ10nm gold-particles against the human Lλ-chain region as described in Methods. Upper left, signal sequence-dependent pVII display with the pGALD7-rescued phagemid (phagemid:helper phage, 1∶107). Lower left, signal sequence-dependent pIX display with the pGALD9-rescued phagemid (phagemid:helper phage, 1∶1). Upper right, signal sequence-independent pVII display with the pGALD7ΔL-rescued phagemid (phagemid:helper phage, 20∶1). Lower right, signal sequence-independent pIX display with the pGALD9ΔL-rescued phagemid (phagemid:helper phage, 261∶1). As depicted in Fig. S2, the phagemid and helper phage virions can easily be distinguished based on either short or long morphology, respectively. Yet only the phagemid encodes the scFv, both short and long virions display the fusion protein. The samples exhibit strong variation in phagemid to helper phage rations (determined by infectious titration) depending on capsid and presence or absence of the signal sequence on the fusion protein. Scale bar: 200nm. (TIF) [file pone.0017433.s003.tif]
