# Supplementary material for: Expanding the Versatility of Phage Display II: Improved Affinity Selection of Folded Domains on Protein VII and IX of the Filamentous Phage
Source: PLoS One. 2011 Feb 24;6(2):e17433. doi: 10.1371/journal.pone.0017433 (PMC3044770; doi:10.1371/journal.pone.0017433)
Supplement: Table S1 — A268nm vs. infection titer input in the “monoclonal” mock selection. (DOC) [file pone.0017433.s004.doc]

**Table S1.** A_268nm_ vs. infection titer input in the “monoclonal” mock selection

| **Experiment** |  | **Display type** | **Input: 10^10^ virions** | **Input: 10^5^ virions** |
| --- | --- | --- | --- | --- |
| **1** | **Input: cfu^ampR^/cfu^kanR^** | pVII | 5.4x 10^9^/3.5x 10^7^ | 5.4x 10^4^/3.5x 10^2^ |
| **1** | **Input: cfu^kanR^/cfu^kanR^** | pVII^pelB^ | 1.8x 10^7^/1.5x 10^10^ | 1.8x 10^2^/1.5x 10^5^ |
| **2** | **Input: cfu^ampR^/cfu^kanR^** | pVII | 4.0x 10^9^/4.7x 10^7^ | 4.0x 10^4^/4.7x 10^2^ |
| **2** | **Input: cfu^kanR^/cfu^kanR^** | pVII^pelB^ | 5.2x 10^6^/1.0x 10^11^ | 52/1.0x 10^6^ |
