# Supplementary material for: Expanding the Versatility of Phage Display II: Improved Affinity Selection of Folded Domains on Protein VII and IX of the Filamentous Phage
Source: PLoS One. 2011 Feb 24;6(2):e17433. doi: 10.1371/journal.pone.0017433 (PMC3044770; doi:10.1371/journal.pone.0017433)
Supplement: Table S2 — Oligonucleotide sequences. (DOC) [file pone.0017433.s005.doc]

**Table S2.** Oligonucleotide sequences

| 1. | **a41g-frwd*** | 5’- AGAGGAGAAATTAACCATGGAATACCTATTGCCTACGGC-3’ |
| --- | --- | --- |
| 2. | **a41g-rev*** | 5- GCCGTAGGCAATAGGTATTCCATGGTTAATTTCTCCTCT-3’ |
| 3. | **pHOG_EcoRI_frwd** | 5’-TAGCTCACTCATTAGGCACCC-3’ |
| 4. | **scTCR_rev** | 5’-TTTGGATCCAGCGGCCGC-3’ |
| 5. | **pVII_EcoRV^†^** | 5’-ATATGATATCAGAATGGAGCAGGTCGCGGATTTCG-3’ |
| 6. | **pVII_NheI^†^** | 5’-ATATGCTAGCTTATCATCTTTGACCCCCAGCGATTATACC-3’ |
| 7. | **pIX_EcoRV^‡^** | 5’-ATATGATATCAGAATGAGTGTTTTAGTGTATTCTTTCGCC-3’ |
| 8. | **pIX_NheI^‡^** | 5’-ATATGCTAGCTTATCATGAGGAAGTTTCCATTAAACGGG-3’ |

* QuikChange™ *in vitro* mutagenesis primers used to changing the first nucleotide in the second codon of the pelB ORF from A to G in pSEX81, thereby creating a new *Nco*I RE site. Following mutagenesis, the vector was *Nco*I digested, re-ligated and used as template in a second PCR retrieving the relevant part of the vector using the primer pair pHOG_EcoRI_frwd/scTCR_rev. This PCR fragment was then moved into the original pSEX81 on the compatible *EcoR*I/*Hind*III RE sites (Fig. 2) creating pSEX81ΔL.

^†^ PCR primer used to amplify the pVII ORF from M13K07.The primers were taggend with *EcoR*V/*Nhe*I RE sites and resulting PCR product moved into pSEX81and pSEX81ΔL on the compatible RE sites (Fig. 2), creating pGALD7 and pGALD7ΔL, respectively.

^‡^ PCR primer used to amplify the pIX ORF from M13K07. The primers were taggend with *EcoR*V/*Nhe*I RE sites and resulting PCR product moved into pSEX81and pSEX81ΔL on the compatible RE sites (Fig. 2), creating pGALD9 and pGALD9ΔL, respectively.
