# Supplementary material for: Expanding the Versatility of Phage Display II: Improved Affinity Selection of Folded Domains on Protein VII and IX of the Filamentous Phage
Source: PLoS One. 2011 Feb 24;6(2):e17433. doi: 10.1371/journal.pone.0017433 (PMC3044770; doi:10.1371/journal.pone.0017433)
Supplement: Table S3 — GenBank accession numbers. (DOC) [file pone.0017433.s006.doc]

**Table S3.** GenBank accession numbers

| **1.** | pGALD7-scFv anti-phOx | HQ528245 |
| --- | --- | --- |
| **2.** | pGALD7ΔL-scFv anti-phOx | HQ528246 |
| **3.** | pGALD7ΔLFN-scTCR Vαβ4B2A1 | HQ528247 |
| **4.** | pGALD9-scFv anti-phOx | HQ528248 |
| **5.** | pGALD9ΔL-scFv anti-phOx | HQ528249 |
| **6.** | pGALD9ΔLFN-scTCR Vαβ4B2A1 | HQ528250 |
